# Unexpected acute kidney injury requiring dialysis after routine pulsed field pulmonary vein isolation: a case report

**DOI:** 10.1093/ehjcr/ytaf669

**Published:** 2025-12-22

**Authors:** Bart A Mulder, Michele F Eisenga, Mark Eijgelsheim, Yuri Blaauw

**Affiliations:** Department of Cardiology, University Medical Centre Groningen, University of Groningen, Hanzeplein 1, 9700RB, Groningen, The Netherlands; Department of Internal Medicine, University Medical Centre Groningen, University of Groningen, Hanzeplein 1, 9700RB, Groningen, The Netherlands; Department of Internal Medicine, University Medical Centre Groningen, University of Groningen, Hanzeplein 1, 9700RB, Groningen, The Netherlands; Department of Cardiology, University Medical Centre Groningen, University of Groningen, Hanzeplein 1, 9700RB, Groningen, The Netherlands

**Keywords:** Atrial fibrillation, Pulsed field ablation, Pulmonary vein isolation, Acute kidney injury, Haemolysis, Case report

## Abstract

**Background:**

Pulmonary vein isolation (PVI) using pulsed field ablation (PFA) has emerged as a promising treatment for atrial fibrillation (AF). However, haemolysis-induced acute kidney injury (AKI) remains a concern, particularly with higher numbers of applications.

**Case summary:**

We report the case of a 62-year-old male with a history of CABG who underwent PVI using a 35 mm pentaspline PFA catheter. Pre-procedural CT imaging revealed a large 37 mm left common PV ostium. Standard energy delivery protocols were followed, including two ‘olive’ applications per vein. The patient was discharged the same day. That evening, he developed dark ‘cola-coloured’ urine. Upon remote consultation the next day, he was advised to increase fluid intake to 3 L that day. His urine colour normalized by the following day, but he experienced a 3 kg weight gain and oliguria and presented to the emergency department. Laboratory tests showed severe AKI (eGFR to 6 mL/min/1.73 m²), necessitating urgent dialysis. Laboratory results showed modest but clinically relevant elevation of haemolysis markers, while urine analysis showed breakdown product of erythrocytes. After several haemodialysis sessions, the patient’s urine output improved, and he was eventually discharged. Two months after the procedure, his renal function recovered to normal values.

**Discussion:**

While prior experience suggests that limiting PFA applications to fewer than 70 may reduce the risk of haemolysis-associated renal injury, this case demonstrates that even with 40 applications and minimal systemic haemolysis, AKI requiring dialysis can occur. This highlights the need for heightened vigilance, early fluid management, and prompt intervention to support renal recovery after PFA.

Learning points

- Even moderate PFA application numbers can result in significant renal injury under certain conditions during PVI.
- Left atrial anatomy, particularly large common ostia, may increase the risk of localized red blood cell electroporation, especially when catheter contact is suboptimal.
- Early recognition, aggressive fluid management, and timely nephrology consultation are critical to support renal recovery.

## Introduction

Pulmonary vein isolation (PVI) remains the cornerstone of rhythm control therapy for atrial fibrillation (AF).^1^ Pulsed field ablation (PFA) has rapidly transformed the field by offering a safe and efficient means of achieving PVI.^2–4^ Notably, some of the severe complications associated with traditional thermal ablation methods, such as atrioesophageal fistula and phrenic nerve injury, have not been reported with PFA or are only observed as transient events.^5,6^ However, new complications have emerged. One such complication is acute kidney injury (AKI) secondary to haemolysis, which has been reported in rare cases.^7^ In the MANIFEST-17K registry, this occurred in ∼0.03% of patients, often associated with delivery of excessive number of PFA pulses.^8^ Here, we present a case of routine PFA-based PVI complicated by mild haemolysis-induced AKI requiring temporary dialysis.

## Summary figure

**Figure:**
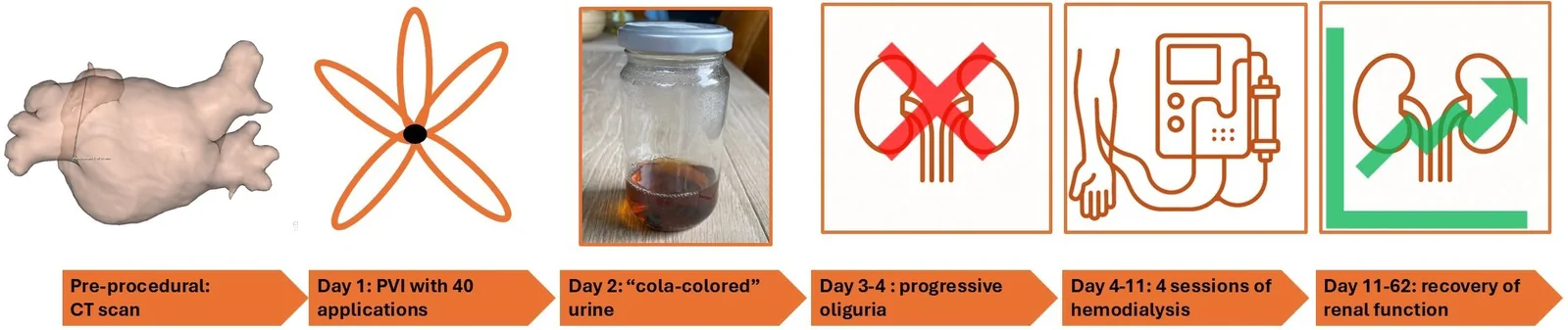


## Case presentation

**Figure 1:**
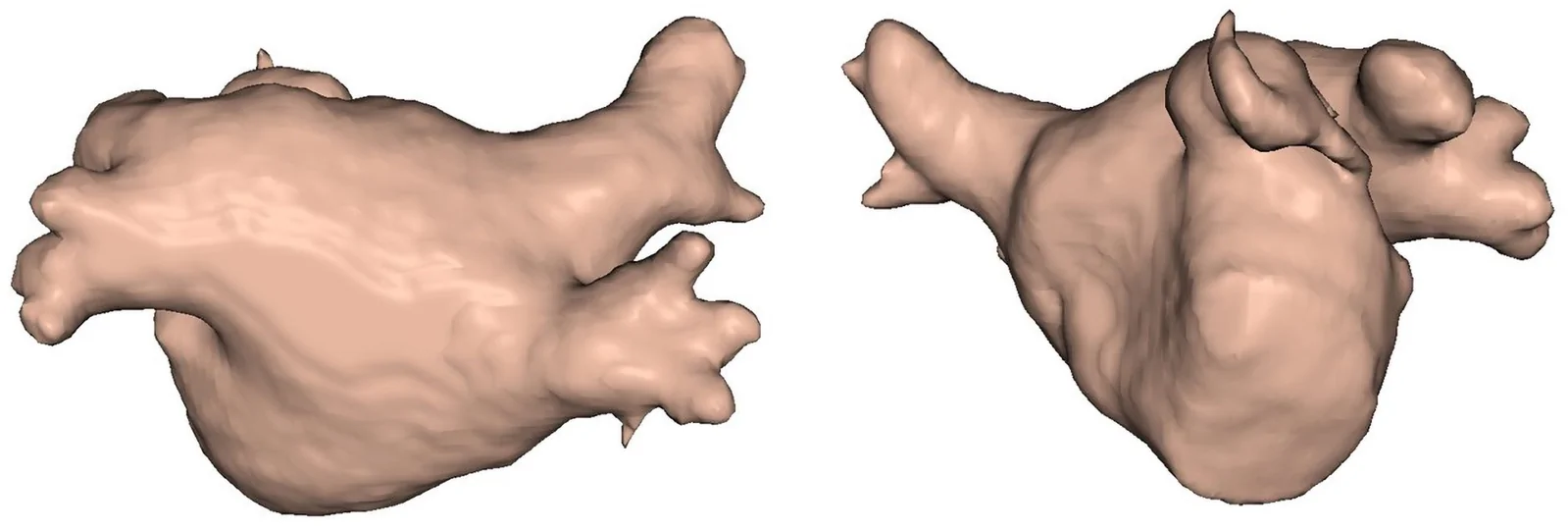
3D reconstruction of the left atrium illustrating a prominent left-sided common pulmonary vein ostium.

A 62-year-old male with a history of coronary artery bypass grafting (CABG) 1 year prior was referred to our hospital for PVI due to symptomatic paroxysmal AF. His medical history was otherwise unremarkable, with no history of kidney disease. He had a body mass index (BMI) of 26.9, and physical examination revealed no abnormalities. Transthoracic echocardiography (TTE) demonstrated a normal left ventricular ejection fraction with no left atrial enlargement. Pre-procedural computed tomography (CT) imaging revealed a large 37 mm left-sided common ostium and two right-sided pulmonary veins (*Figure 1*). Laboratory investigations on the day of the procedure were within normal limits (eGFR 80 mL/min/1.73 m², creatinine 89 µmol/L).

**Figure 2:**
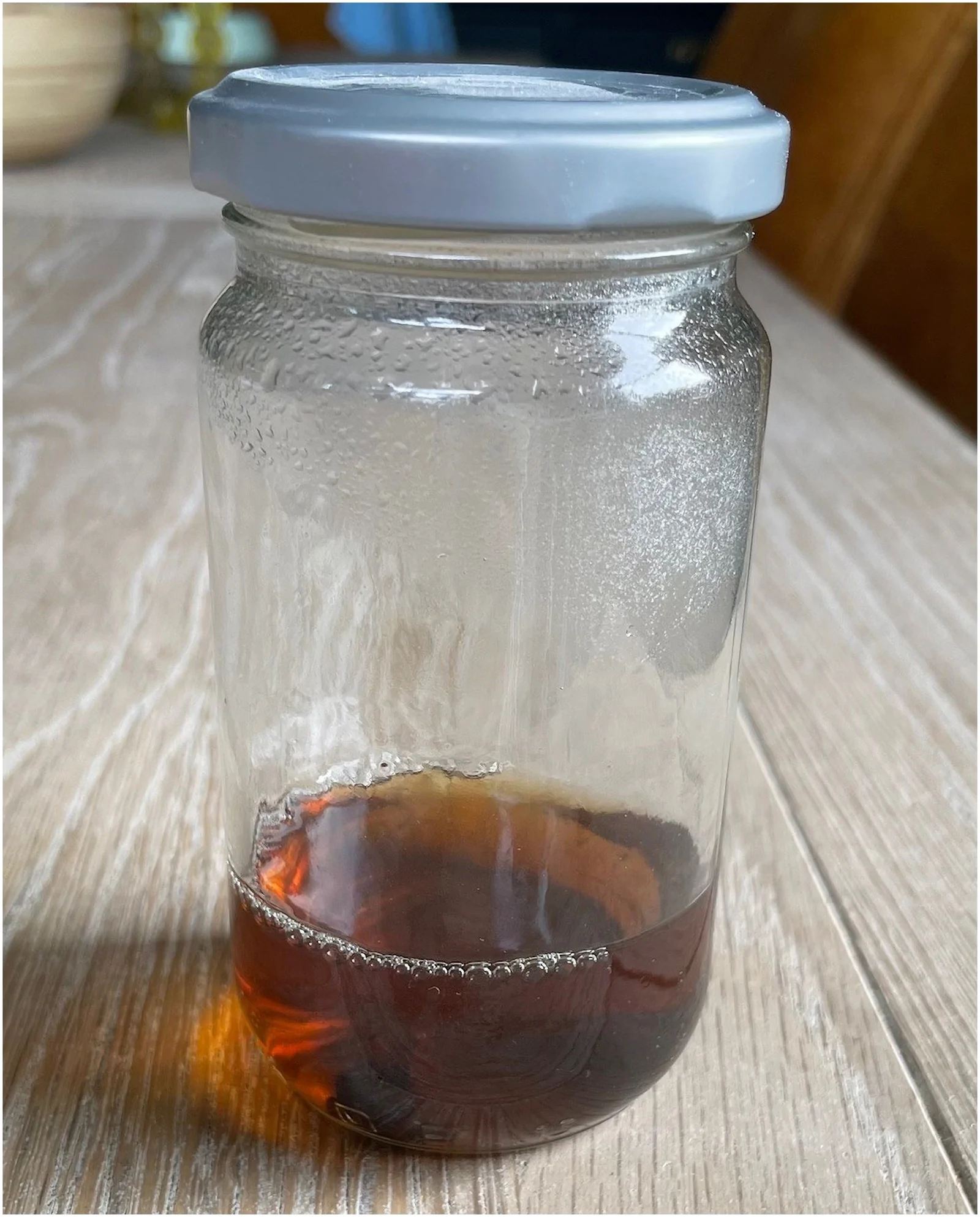
Cola-coloured urine 1 day after PVI.

**Figure 3:**
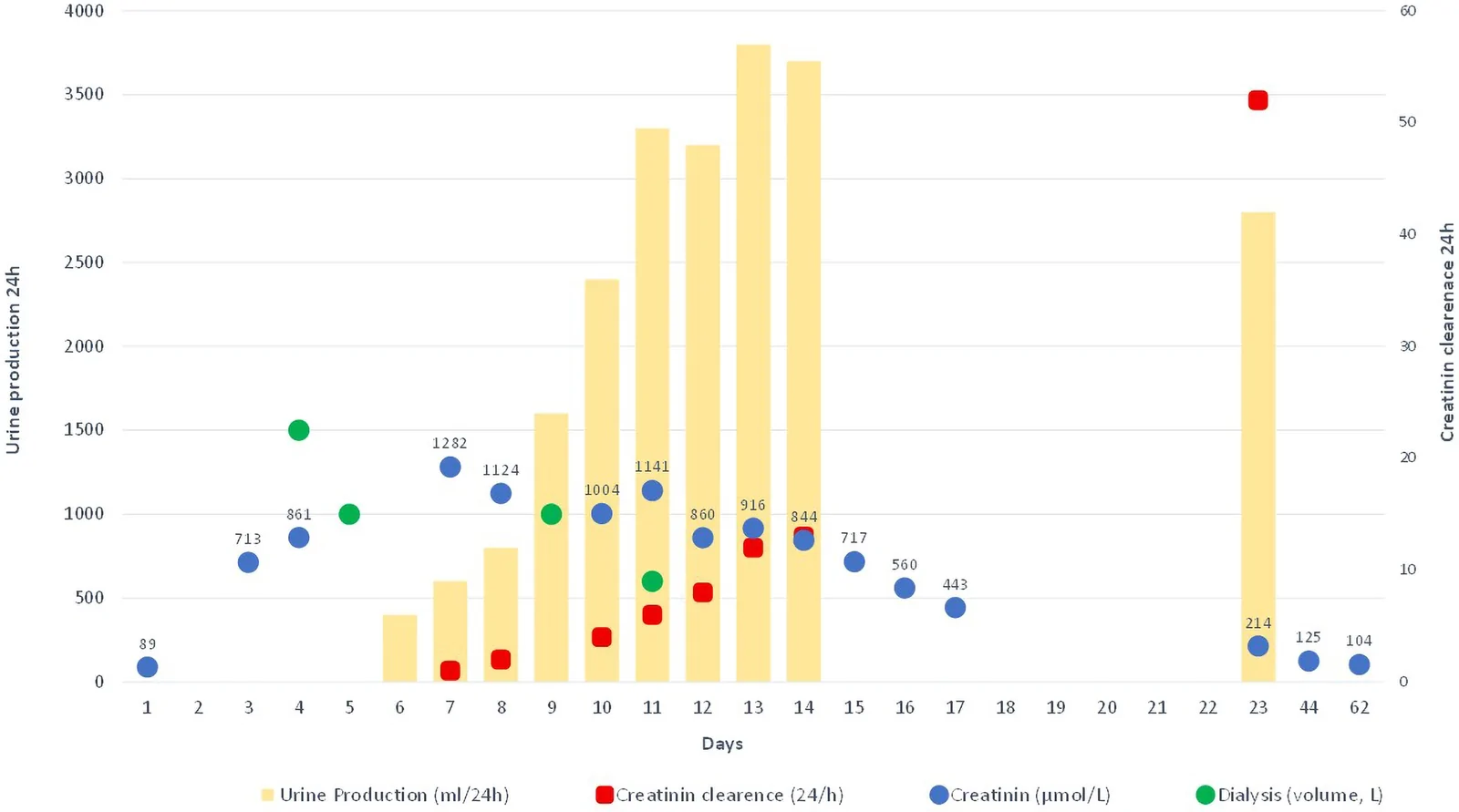
Changes in serum creatinine, creatinine clearance, and urine output following PVI.

A PVI procedure was performed using a 35 mm pentaspline Farapulse catheter under deep sedation with propofol and esketamine. The catheter size was selected since a large common ostium was observed on pre-procedural CT (*Figure 1*). Vascular access was obtained via two femoral vein punctures at 09:33, followed by a transseptal puncture at 09:35. Atropine 0.5 mg was administered immediately thereafter. Standard applications were delivered according to institutional protocol, including two ‘olive’ applications per vein.^9^ In total 40 applications were performed and all pulmonary veins were successfully isolated. Intracardiac echocardiography was used to confirm adequate catheter-tissue contact throughout the procedure. The sheaths were removed at 10:05. Activated clotting time (ACT) was measured once during the procedure and was >400 s. Haemodynamic parameters remained stable throughout the procedure: baseline blood pressure was 109/75 mmHg, the lowest recorded value (prior to the first energy application) was 95/73 mmHg, and at the end of sedation, blood pressure had normalized to 114/83 mmHg. Post-procedure, the patient recovered well on the ward and was discharged at 17:18 h the same afternoon. However, later that evening, he developed cola-coloured urine (*Figure 2*) and contacted the emergency department at 22:30 h. The attending resident was initially unavailable and attempted to return the patient’s call later but was unable to reach him. The following morning, the patient re-established contact and was advised to increase oral fluid intake to at least 3 L per day and to monitor for any worsening of symptoms. On follow-up the next day, the patient reported that the urine colour had normalized but that his urinary output had decreased, and he felt generally unwell. He had also gained 3 kg in body weight. He was advised to present to the emergency department, where laboratory evaluation revealed acute kidney injury (*Table 1*). Laboratory tests revealed an acute rise in serum creatinine, mildly elevated lactate dehydrogenase (LDH), and normal haptoglobin levels (measured once). Renal ultrasound excluded obstructive uropathy and demonstrated normal kidney size and echogenicity. Urinalysis revealed a few pigmented granular casts. Initial management included supportive care with intravenous fluids (3 L). Loop diuretics (bumetanide 5 mg twice daily) were initiated for progressive oliguria in an attempt to stimulate diuresis but were discontinued once anuria was established and the patient ultimately required temporary haemodialysis. The patient underwent a total of four haemodialysis sessions, primarily for ultrafiltration of approximately 600–1400 mL per session (*Figure 3*). One week after the procedure, spontaneous urine output resumed, and he entered a polyuric phase. Multiple 24 h urine collections during the admission showed return of creatinine clearance. Two weeks after admission, he was discharged without the need for further dialysis. By 2 months after admission, renal function had returned to baseline values (eGFR 66 mL/min/1.73 m², creatinine 104 µmol/L).

**Table 1:** Blood and urine test results day of procedure and 2 days later at the emergency department

	Day of PVI	2 days after PVI	Expected in haemolysis
Haemoglobin (mmol/L)	9,9	8,2	↓ or stable
Potassium (mmol/L)	4,1	5,0	↑
Creatinine (µmol/L)	89	713	↑ (if AKI)
Urea (mmol/L)	6,0	21,3	↑
eGFR (mL/min/1.73 m²)	80	6	↓
Troponin (ng/L)	—	3666	→
Haptoglobin (g/L)		0,7	↓↓
CK (U/L)		409	→ or mild ↑
ASAT (U/L)		53	↑
ALAT (U/L)		25	→
Alkaline phosphatase (U/L)		46	→
GGT (U/L)		26	→
Total bilirubin (µmol/L)		10	↑ (indirect)
Peripheral blood smear			
Elliptocytes		Present (+)	Sometimes ↑ in haemolysis
Fragmentocytes		Not observed	↑ in microangiopathic haemolysis
Urinalysis (dipstick and microscopy)			
Haemoglobin		3+	↑ (haematuria or haemolysis)
Protein		2+	↑ (possible glomerular damage)
Erythrocytes per high-power field		Numerous	=^a^
Dysmorphic erythrocytes (%)		10–40%	=^a^
Acanthocytes (%)		2%	=^a^
Granular casts		Few	May indicate tubular injury

^a^Would suggest glomerular bleeding through damaged glomerular basement membrane.

## Discussion

PFA-induced haemolysis-associated renal injury requiring dialysis is a rare complication, reported in ∼0.03% of patients and typically associated with extensive energy delivery (>100 applications in seven cases).^7,8^ A recent published case showed severe haemolysis and acute kidney failure without the need for dialysis with 48 applications.^10^ The present case underscores the variability in clinical response, as acute kidney injury requiring dialysis occurred despite the use of only 40 applications for PVI, well below the numbers previously reported in similar cases.

Previous studies have shown that mild haemolysis is relatively common following PFA, with depleted haptoglobin levels observed in up to 28% of patients.^7^ In contrast, this patient had normal haptoglobin levels, and signs of haemolysis were limited to dark-coloured urine. Other haemolysis-related biomarkers were only mildly abnormal. The pathophysiology of pigment nephropathy in haemolysis is classically dose-dependent: high levels of free haemoglobin overwhelm haptoglobin-binding capacity, leading to renal filtration and uptake by the proximal renal tubule. Within the proximal tubule, free haemoglobin is catabolized into free heme and ferrous iron, which, in excess, induce oxidative injury and tubular cell death. Once proximal tubular reabsorption capacity is overwhelmed, free haemoglobin reaches downstream segments, where it dissociates into free heme that binds uromodulin, forming cytotoxic, obstructive casts. This results in oxidative stress, tubular obstruction, and direct cytotoxicity.^11^ Several additional factors can contribute to this process, such as pre-existing renal dysfunction or use of nephrotoxic medications, but these factors were not present in this case.

**Figure 4:**
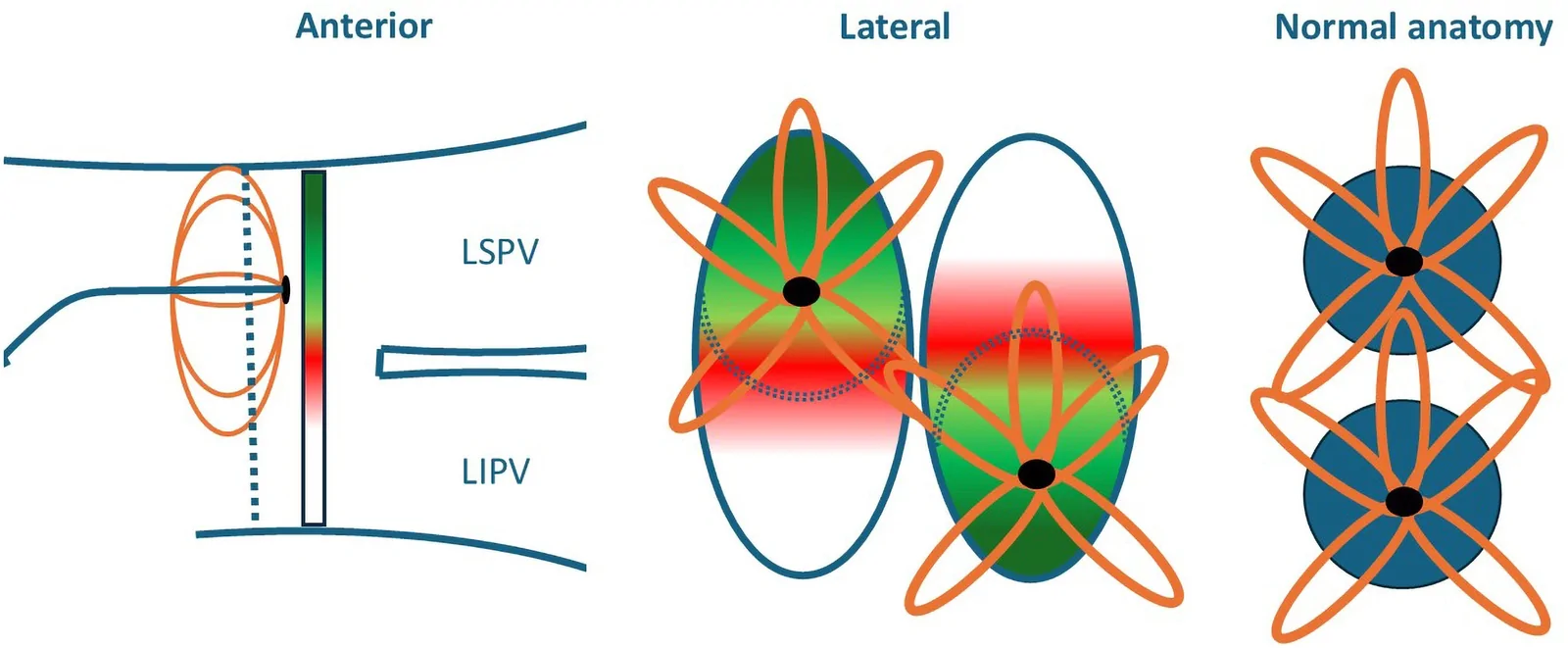
Relationship between optimal catheter positioning (dotted line) and ineffective contact with the pulmonary vein ostium.

One plausible explanation involves patient-specific anatomy, notably a large left-sided common pulmonary vein ostium. This anatomical variant may impair circumferential contact between the pentaspline PFA catheter and the atrial tissue. Poor contact in certain regions could result in non-target tissue electroporation, particularly of blood elements.^12^ If a portion of the catheter delivers energy into circulating blood rather than atrial myocardium, localized red blood cell lysis may occur even in the absence of widespread systemic haemolysis (*Figure 4*). When ablation occurs without consistent tissue contact, portions of the catheter may deliver energy directly into the circulating blood pool rather than the atrial wall, leading to localized haemolysis. This process may occur without being consistently reflected in systemic laboratory markers such as haptoglobin or LDH, but still sufficient to cause renal injury especially if renal perfusion is compromised. The patient’s volume status may therefore have been a contributing factor.^13^ Patients undergoing PVI are often kept fasting and may be relatively fluid-depleted at the time of the procedure. This mild hypovolaemia can impair renal perfusion and reduce the kidney’s ability to clear free haemoglobin, thereby increasing the risk of pigment nephropathy. In this case, even though the total number of applications was within a moderate range, the combination of possible suboptimal catheter-tissue contact in a large common ostium and a potentially low intravascular volume state may have contributed to red blood cell lysis and impaired haemoglobin clearance; however, it cannot be excluded that additional, as yet unidentified, factors also played a role.^14^

This case emphasizes the importance of individualized risk assessment and preventive strategies, particularly in anatomically complex patients undergoing PFA. Further studies are needed to assess whether periprocedural hydration protocols, contact-force feedback, or real-time lesion assessment tools could reduce the risk of this rare but serious complication.

## Conclusion

PFA-induced haemolysis-associated renal injury is a rare complication, typically reported in cases involving excessive energy applications. However, this case demonstrates that AKI requiring dialysis can occur even with a moderate number of applications and minimal evidence of systemic haemolysis. It underscores the importance of maintaining a high index of suspicion, initiating early fluid resuscitation, and ensuring prompt nephrology involvement to support renal recovery following PFA.

## Lead author biography


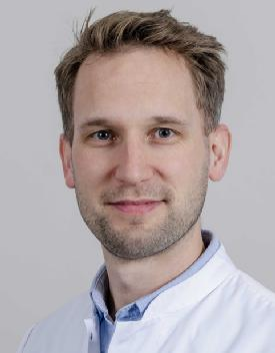


Dr. Bart Mulder is a Dutch cardiac electrophysiologist at the University Medical Center Groningen. His research focuses on clinical studies of atrial fibrillation ablation and the evaluation and implementation of novel ablation technologies.

## Data Availability

The data underlying this article will be shared on reasonable request to the corresponding author.

## Notes

**Consent:** The authors confirm that written consent for submitting and publishing this case report, including images and associated text, has been obtained from the patient in line with the COPE guidance.
