# Correction to: Unexpected acute kidney injury requiring dialysis after routine pulsed field pulmonary vein isolation: a case report

**DOI:** 10.1093/ehjcr/ytag608

**Published:** 2026-09-02

This is a correction to: Bart A Mulder, Michele F Eisenga, Mark Eijgelsheim, Yuri Blaauw, Unexpected acute kidney injury requiring dialysis after routine pulsed field pulmonary vein isolation: a case report, *European Heart Journal - Case Reports*, Volume 10, Issue 1, January 2026, ytaf669, https://doi.org/10.1093/ehjcr/ytaf669

In the originally published version of this manuscript, there was an error within Table 1 under the ‘Day of PVI’ heading.

The entry for Haemoglobin (mmol/L) has been corrected to read “9,9” instead of “7,5”.

